# Cytological techniques to analyze meiosis in *Arabidopsis arenosa* for investigating adaptation to polyploidy

**DOI:** 10.3389/fpls.2013.00546

**Published:** 2014-01-03

**Authors:** James D. Higgins, Kevin M. Wright, Kirsten Bomblies, F. Chris H. Franklin

**Affiliations:** ^1^School of Biosciences, The University of BirminghamBirmingham, UK; ^2^Department of Organismic and Evolutionary Biology, Harvard University, CambridgeMA, USA

**Keywords:** *Arabidopsis arenosa*, polyploidy, cytology, immunolocalization, meiosis, recombination, synaptonemal complex

## Abstract

*Arabidopsis arenosa *is a close relative of the model plant *A. thaliana*, and exists in nature as stable diploid and autotetraploid populations. Natural tetraploids have adapted to whole genome duplication and do not commonly show meiotic errors such as multivalent and univalent formation, which can lead to chromosome non-disjunction and reduced fertility. A genome scan for genes strongly differentiated between diploid and autotetraploid *A. arenosa* identified a subset of meiotic genes that may be responsible for adaptation to polyploid meiosis. To investigate the mechanisms by which *A. arenosa* adapted to its polyploid state, and the functionality of the identified potentially adaptive polymorphisms, a thorough cytological analysis is required. Therefore, in this chapter we describe methods and techniques to analyze male meiosis in *A. arenosa,* including optimum plant growth conditions, and immunocytological and cytological approaches developed with the specific purpose of understanding meiotic adaptation in an autotetraploid. In addition we present a meiotic cytological atlas to be used as a reference for particular stages and discuss observations arising from a comparison of meiosis between diploid and autotetraploid *A. arenosa*.

## INTRODUCTION

Over the past 15 years, *Arabidopsis thaliana* has prevailed as the model organism of choice to investigate meiosis in plants. It is amenable for such studies due to its annotated genome sequence, collection of mutants, ease of transformation, and established cytology (The Arabidopsis Genome Initiative, 2000). This has led to the identification and characterisation of numerous genes involved in crossover pathways (reviewed by Osman et al., 2011). It has proved extremely successful for understanding diploid meiosis but is limited when investigating the evolution of polyploidy in plants. *A. thaliana* neotetraploids have been synthesized using colchicine to study early responses to genome duplication (Santos et al., 2003). However, established natural autotetraploid populations are not known, hence this species cannot be used to study longer-term adaptation to whole genome duplication. *A. arenosa* is a related species within the genus (Al-Shehbaz and O’Kane, 2002). It is self-incompatible with extant diploid (2*n* = 2x = 16) and established autotetraploid (2*n* = 4x = 32) populations (Koch and Matschinger, 2007; Schmickl et al., 2012). A comparative genome sequence analysis of established populations of autotetraploid *A. arenosa* (Hollister et al., 2012) as well as diploid versus autotetraploid populations have identified a subset of meiotic genes that are strongly differentiated and may be responsible for adaptation to polyploid meiosis (Yant et al., 2013).

To investigate the mechanisms by which *A. arenosa* has adapted to its autopolyploid state, and to verify the functionality of the identified polymorphisms, a thorough cytological analysis is required. Previous cytological studies have focused on characterization of *A. arenosa* polyploids using fixed material (Comai et al., 2003; Carvalho et al., 2010). Here we provide a detailed description of immunocytological techniques as well as optimum plant growth conditions using fresh material. In addition, we provide a comparative cytological meiotic atlas for identifying particular stages of both diploid and tetraploid *A. arenosa*. We also present metaphase I chromosome spreads as examples of fluorescence *in situ* hybridization (FISH) using repetitive DNA probes in this species. Finally, we discuss specific observations from these experiments. This will enable characterization of key meiotic proteins and processes underpinning stable chromosome transmission in *A. arenosa.*

## MATERIALS

### PLANTS

Sow individual diploid and tetraploid *A. arenosa *seeds in 10 cm diameter pots using (50% Sunshine Mix #4/50% fine vermiculite) and grow with 16 h long-day light cycles at 22°C and 8 h dark cycles at 12°C. Particular tetraploid genotypes such as Kasparstein and Trencin require vernalizing (~6 weeks at 4°C), but otherwise seeds will germinate in contact with the soil and moisture. If the seeds have been collected directly from the wild it is necessary to surface sterilize them according to Hollister et al. (2012). From germination it takes ~6–8 weeks for the most rapid accessions to flower, and is subject to genotypic variability. Once growing the plants can be maintained to a year or more by regular re-potting and partial shading. To obtain seeds from these plants it is necessary to cross-pollinate individuals. This can be achieved simply by juxtaposing flowers from different plants so that the pollen is transferred to the style of the opposing plant or using a paintbrush to transfer pollen from plant-to-plant.

### IMMUNOCYTOLOGICAL REAGENTS

(1) Digestion medium: 0.1 g (0.4% w/v) cytohelicase (Sigma C C8274) is dissolved with 0.375 g (1.5% w/v) sucrose and 0.25 g (1% w/v) polyvinylpyrrolidone (Sigma MW 40,000) in 25 ml sterile deionized water. Aliquots of 1 ml are dispensed and can be stored at -20°C.
(2) Spreading medium: 1.0–1.5% (v/v) Lipsol (Appleton Woods LP40023) in sterile deionized water.
(3) Paraformaldehyde fixative: Weigh out 4 g paraformaldehyde (EM grade) in the fume hood. Dissolve in 100 ml of sterile deionized water pre-warmed to 60°C and add four drops of 1 M NaOH. Stir the mixture on a magnetic stirrer for 1 h, or until it dissolves before filtering through Whatman paper. Adjust the pH to 8.0. The fixative can be stored for up to 1 week at 4°C.
(4) Blocking buffer: Make 1% (w/v) bovine serum albumin (BSA) in 1× phosphate buffer saline (PBS; we tend to use prepared tablets, but PBS can also be made up from 10× stock with 1.37 M NaCl, 27 mM KCl, 100 mM Na_2_HPO_4_, 18 mM KH_2_PO_4,_adjusted to pH 7.4 if necessary) and add Triton X-100 for a final volume of 0.1% (v/v). Autoclave before storage at room temperature. The working solution of the buffer is 1:10 with sterile deionized water.
(5) Primary antibodies: Make up to the relevant dilution, e.g., 1:100, 1:500, 1:1000 in blocking buffer.
(6) Washing solution: 1× PBS with 0.1% (v/v) Triton X-100.
(7) Secondary antibodies: Make up to the relevant dilution (generally 1:50/1:200) in blocking buffer, e.g., anti-rat, anti-rabbit, anti-guinea pig, anti-mouse, conjugated to fluorescein isothiocyanate (FITC), Cy3, or Alexa Fluor dyes^1^
(8) Counterstaining solution: Prepare 4′,6-diaminido-2-pheny-lindole (DAPI) as a stock solution at 1 mg/ml in sterile deionized water. Dispense in aliquots and store at -20°C. For working solution add 10 μl of 1 mg/ml stock solution to 1 ml anti-fade mounting medium such as Vectashield^2^

### CYTOLOGICAL REAGENTS

(1) Fixative: Mix three parts of absolute ethanol with one part glacial acetic acid. Prepare fresh each time.
(2) 0.01 M Citrate Buffer: Prepare a working solution of the buffer (pH 4.5) by using 4.45 ml 0.1 M sodium citrate and 5.55 ml 0.1 M citric acid, made up to 100 ml with sterile deionized water.
(3) Stock digestion medium: Dissolve 1% (w/v) cellulase (Sigma C1794), 1% (w/v) pectolyase (Sigma P5936) in a working solution 0.01 M citrate buffer, pH 4.5. Store in aliquots at -20°C.
(4) Digestion medium: mix 333 μl of the stock digestion medium with 667 μl 0.01 M citrate buffer, pH 4.5.
(5) 65% (v/v) acetic acid: Dilute glacial acetic acid with sterile deionized water.
(6) Counterstaining solution. See *Immunocytological Reagents (8)*.

### REAGENTS FOR FLUORESCENCE *IN SITU* HYBRIDIZATION

(1) Probe labeling: Use the nick translation labeling kit^3^ following the manufacturer’s instructions. Use either biotin-16-deoxyuridine triphosphate (dUTP) or digoxigenin-11-dUTP as nucleotide conjugates for DNA labeling.
(2) Hybridization mix: Weigh out 1 g dextran sulfate (use high MW 500,000), 5 ml deionized formamide, and 1 ml 20× saline-sodium citrate (SSC) made up to 7 ml with sterile deionized water. Dissolve at 65°C, cool and pH to 7.0. Aliquot this into 1.5 ml microfuge tubes and store at -20°C.
(3) Prepare 20 μl of probe mixture per slide; 14 μl of hybridization mix, 0.5–2 μl of labeled probe, and if necessary add sterile deionized water for a final volume of 20 μl. Thus, the final hybridization mix consists of 50% (w/v) deionized formamide, 2× SSC and 10% (w/v) dextran sulfate and adjust pH to 7.0.
(4) Vulcanizing rubber solution: (e.g., as found in bicycle tire repair kits).
(5) Make up post-hybridization washes: Three Coplin jars of 50% (w/v) formamide-2× SSC pH 7.0, (150 ml deionized formamide, 30 ml 20× SSC and 120 ml sterile deionized water), with 4T buffer [4× SSC + 0.05% (v/v) Tween 20] for all subsequent washes.
(6) For detection of digoxigenin probes, make up antibodies as either anti-digoxigenin-FITC or anti-digoxigenin-rhodamine at 5 ng/μl in digoxigenin blocking solution shortly before use. The blocking solution is made with 4T buffer and 0.5% Roche Digoxigenin blocking reagent, centrifuged at 19,000 g for 5 min and the supernatant stored in 1 ml aliquots at -20°C.
(7) For biotin labeled probes use Streptavidin-Cy3/FITC^4^ made up in biotin blocking solution. This is made with 4T buffer and 5% (w/v) dried skimmed milk. Centrifuge at 19,000 g for 5 min and store the supernatant in 1 ml aliquots at -20°C.

### DNA PROBES FOR FLUORESCENCE *IN SITU* HYBRIDIZATION

(1) Telomere probe. Oligonucleotide sequences 5′-TTTAGGGTTT AGGGTTTAGGGTTTAGGGTTTAGGG-3′ and 5′-CCCTAAACC CTAAACCCTAAACCCTAAACCCTAAA-3′ (100 pmol each) were used in a 50 μl primary reaction as primer and template. The polymerase chain reaction (PCR) was: denaturation at 93°C for 30 s; annealing at 55°C for 45 s; extensión at 72°C, 45 s; 30 cycles. A secondary PCR using 1 μl template from the primary PCR but replacing deoxythymidine triphosphate (dTTP) with dUTP-biotin/digoxigenin was used to label the probe. The probe labeled with biotin can be detected using streptavidin conjugated to a fluorophore and the digoxigenin probe can be detected using an anti-digoxigenin antibody conjugated to a fluorophore.
(2) Centromere probe. Oligonucleotide sequences 5′-AGCTTCTT ATTGCTCTCAACGG-3′ and 5′-TTAGAAGCTCCAAAACCGAAAA-3′were used to amplify DNA extracted from *A. arenosa*. The PCR reaction was: denaturation at 93°C for 2 min followed by 30 cycles of 93°C for 25 s; annealing at 57°C for 30 s; extension at 72°C for 40 s according to Comai et al. (2003).
(3) 5*S *ribosomal DNA probe. Plasmid pCT4.2 containing the 5*S* rDNA gene wfrom *A. thaliana* as a 500 bp insert cloned in pBlu. Modified DNA base analogs can be incorporated using either the biotin or DIG Nick translation kit according to manufacturers’ instructions^5^.
(4) 45*S *ribosomal DNA probe. Clone pTa71 (Gerlach and Bedbrook, 1979) containing a 9-kb *Eco*RI fragment of *Triticum aestivum* consisting of the 18*S*–25*S* rRNA genes and the spacer regions. Incorporate modified DNA base analogs as in (3).

## METHODS

### PLANT MATERIAL

For undertaking an analysis of *A. arenosa* male meiosis, the correct material has to be collected. In **Table 1**, bud sizes have been measured from tip to base using a calibrated graticule (so that 10 bars = 1 mm) within the eyepiece of a binocular dissecting microscope. Meiotic stages were then determined using at least five replicates from fresh material (see immunolocalization) and fixed material (see cytological preparation).

**Table 1:** Bud sizes and meiotic stages in diploid and tetraploid *A. arenosa.*

Bud size (mm)	Diploid (meiotic stage)	Tetraploid (meiotic stage)
0.65	G2	G2
0.7	G2 and leptotene	G2 and leptotene
0.75	Leptotene	Leptotene and zygotene
0.8	Leptotene–pachytene	Leptotene and zygotene
0.85	Diakinesis–tetrad	Zygotene and pachytene
0.9	Diakinesis–tetrad	Zygotene–MI
0.95	Metaphase I–tetrad	Pachytene–MII
1.0	Metaphase I–pollen	Pachytene–tetrad
1.1	Pollen	Tetrad
1.2	Pollen	Pollen

### IMMUNOLOCALIZATION

(1) Collect fresh inflorescences using Watchmaker’s forceps and place onto moistened filter paper in a Petri-dish.
(2) Excise the anthers from the buds of the correct size using a dissecting microscope, Watchmaker’s forceps, and a fine mounted needle. For each slide, anthers from a few buds (~2–4) may be used for meiotic stages G2 to leptotene and for zygotene to pachytene, ~1–2 buds.
(3) For anthers from buds < 0.8 mm it is advised to transfer directly to a pre-cleaned slide with a drop (5 μl) of digestion medium (for buds > 0.8 mm go to point 3.2.8).
(4) The anthers can then be macerated with a small brass rod or equivalent blunt object by tapping for a minute and then a second 5 μl digestion medium is added. This is to mechanically break the anther cell walls so the meiocytes may be released into the digestion medium.
(5) Transfer the slide to a moist box at 37°C for 2 min to aid cell wall disruption.
(6) Then remove the slide, add 10 μl 1% Lipsol and spread cells with the mounted needle (to remove cytoplasm from the chromosomes).
(7) Add 20 μl 4% paraformaldehyde to the digested cell mixture on the slide, mix with the pipette tip and then allow to air dry in a fume-hood for > 1 h (go to step 3.2.12).
(8) For anthers > 0.8 mm, add 2 μl sterile distilled water onto a cavity slide.
(9) Add the anthers and cut transversely with a razor blade (or scalpel) and squeeze out the meiocytes with a thick (1 mm ø) mounted needle or brass rod.
(10) Add 8 μl digestion medium to the meiocytes, mix with the mounted needle and transfer to a moist box at 37°C for 3–4 min.
(11) Remove digested meiocytes without anther debris using a yellow tip cut off at the end with a pair of scissors and add to a pre-cleaned slide.
(12) Add 10 μl 1.0–1.5% Lipsol and gently spread with a fine mounted needle. Note: 1.5% Lipsol is recommended for pachytene spreads to remove cytoplasm and reduce background.
(13) Add 20 μl 4% paraformaldehyde and mix with a pipette tip, then allow to air dry >1 h in a fume-hood.
(14) Add 50 μl blocking solution containing primary antibodies at a preferred concentration directly to the slide.
(15) Cover slides with parafilm by placing on top and incubate at 37°C for 30 min or overnight at 4°C in a sealed plastic container with damp tissue paper to prevent desiccation.
(16) After the incubation, wash slides in washing solution (2 × 5 min).
(17) Drain off excess wash buffer by standing on tissue paper for ~2 min.
(18) Add secondary antibodies and incubate at 37°C for 30 min (as step 3.2.15).
(19) Then wash as in step 3.2.16.
(20) Drain off excess washing solution by standing on tissue paper for ~2 min and then add an appropriate mounting medium, e.g., DAPI in Vectashield.

### CYTOLOGICAL PREPARATION

(1) Fix buds in (5–20 ml) 3:1 (v/v) ethanol:acetic acid solution.
(2) Add fresh fixative solution after 1 h and then leave > 24 h.
(3) Whole buds may be used successfully, but for better spread preparations it is advisable to dissect the anthers from the buds using Watchmaker’s forceps and a fine mounted needle.
(4) Wash dissected buds/anthers with citrate buffer by adding 500 μl and then removing (2 × 5 min).
(5) Add the cell wall digesting enzymes prepared in citrate buffer (500–1000 μl) to the washed material and ensure material is submerged using Watchmakers forceps.
(6) Incubate in a humidified atmosphere, e.g., a sandwich box containing damp tissues at 37°C (50 min for anthers and 75 min for buds).
(7) After incubation, remove the cell wall enzyme digesting solution and replace with cold (4°C) ~0.5 ml sterile distilled water to stop the enzymatic reaction and prevent over-digestion.
(8) (8) Place one bud or a few anthers (5–10) onto a slide, with a small drop of water (~2 μl) and then quickly macerate with a mounted needle, ensuring that the material does not dry out.
(9) Add 7 μl 65% acetic acid to the cells, place on a hot-plate at 45°C and spread the mixture using the mounted needle and then leave ~30 s.
(10) Then place the slide on the bench and add 2× 200 μl 3:1 fixative as a ring around the material. Then pour off the excess fixative, blot with tissue paper and then dry with a commercial hair dryer by warming the back of the slide.
(11) The slides are now ready for basic cytology and can be visualized with a fluorescence microscope after mounting with 7 μl DAPI in Vectashield and adding covering with a cover-slip.

### FLUORESCENCE *IN SITU* HYBRIDIZATION

(1) Prepare required probes either by incorporating modified DNA base analogs using PCR or by nick translation (as described in Materials).
(2) Add 20 μl of the probe mixture to a slide previously prepared containing spread meiotic chromosomes. Add a cover-slip (22 mm × 22 mm) and then seal edges with vulcanizing rubber solution.
(3) Denature the probe and chromosomal DNA by heating slides on a hot-plate at 70–75°C for 4 min.
(4) Hybridize the probe and chromosomes by incubating overnight at 37°C in a sealed plastic container with damp tissue paper.
(5) Carry out post-hybridization washes by removing the rubber solution and cover-slip either by gloved fingers or using forceps. Wash the slides three times in 50% formamide-2× SSC at 45°C for 5 min for each wash, then once in 2× SSC at 45°C for 5 min, and once in 4T buffer at room temperature for 5 min.
(6) Perform a secondary labeling reaction for probe visualization. Fluorescent secondary antibodies such as anti-digoxigenin or Streptavidin (for biotin) conjugated with fluorochromes such as Cy3, rhodamine and FITC are used according to the manufacturers’ instructions. They are diluted in the immunolocalization blocking buffer (or specific biotin or digoxigenin blocking buffers described in *Materials*) and 50 μl per slide is added, covered with parafilm and incubated in a sealed plastic container with damp tissue paper at 37°C in the dark for 30 min. The parafilm is then removed and the preparations are washed in the washing solution 3 × 5 min.
(7) Slides are then counterstained with 7 μl DAPI in Vectashield.
(8) The FISH preparations can now be viewed with a fluorescence microscope containing filters for DAPI, texas red, Cy3, and FITC and equipped with an image capture and analysis system.

## RESULTS AND DISCUSSION

In developing the methods described in this chapter, several observations have been made when comparing diploid and tetraploid meiosis in *A. arenosa*. First of all, bud sizes were the same for early meiotic stages (G2-leptotene) in the diploid and tetraploid (**Table 1**). However, whilst pachytene is only observed in 0.8 mm buds in the diploid, in the tetraploid it is present in a range of larger buds (0.85–1.0 mm). Moreover, in the diploid, anther size is a reliable indicator of meiotic stage whereas in the tetraploid, meiotic stages and anther sizes are variable. This reduction in cell stage synchronicity may be the result of a delay in chromosome synapsis, which has previously been observed in the absence of ZYP1 (Higgins et al., 2005). A similar situation has also been observed in the *A. thaliana* *retinoblastoma* mutant *(rbr-2*) where buds that would usually contain only pollen contained earlier meiotic stages, representing a likely delay (Chen et al., 2011). Thus, in *A. arenosa* even though initiation of meiosis occurs in anthers of the same sized buds, the diploid develops pollen within 1.0 mm buds whereas in the tetraploid it is 1.2 mm. These bud sizes may reflect an apparent change in pairing and synapsis between the diploid and tetraploid. It may be hypothesized that the tetraploid has evolved slower progression through meiosis to ensure fidelity of crossovers between homologs. This hypothesis may be tested by carrying out a time-course experiment developed by Armstrong et al. (2003).

**FIGURE 1:**
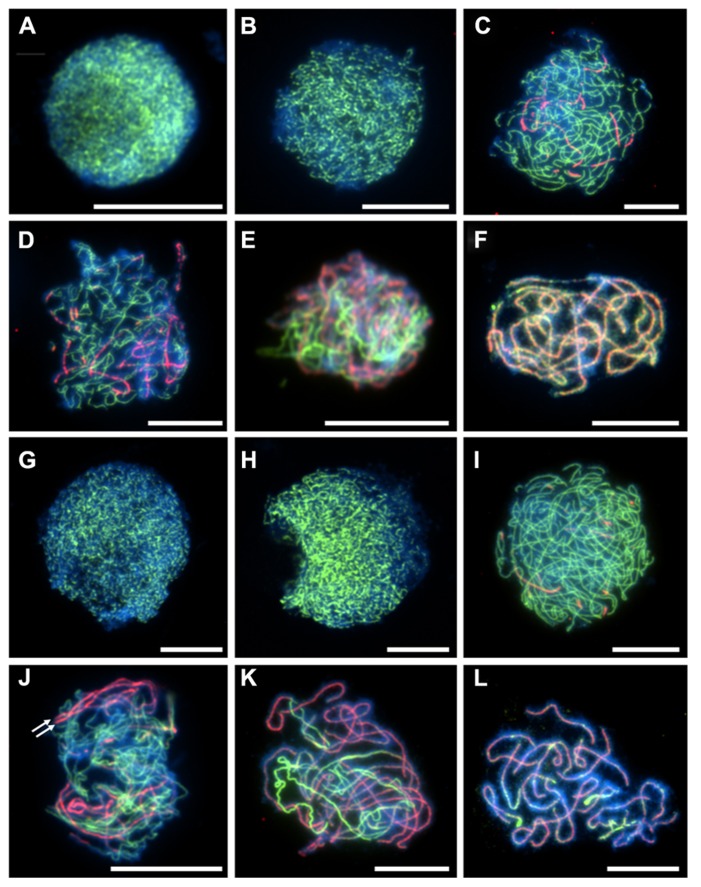
**Immunolocalization of meiotic chromosome axis protein ASY1 (green) and synaptonemal complex transverse filament protein ZYP1 (red) in diploid and tetraploid *Arabidopsis arenosa.*** G2 to pachytene in diploid **(A–F)** and tetraploid **(G–L)**. G2 **(A,G)**, leptotene **(B,H)**, early zygotene **(C,I)**, mid-zygotene **(D,J)**, late-zygotene **(E,K)**, pachytene **(F,L)**. White arrows in **(J)** highlight parallel ZYP1 stretches. Chromosomes have been stained with DAPI (blue) and bar = 10 μm.

The chromosome axis-associated protein ASYNAPSIS1 (ASY1) is required for wild-type levels of inter-homolog recombination, crossover formation, and synapsis in *Arabidopsis* and *Brassica* (Armstrong et al., 2002; Sanchez-Moran et al., 2007). In *A. arenosa*, ASY1 exhibits signatures of selection and strong differentiation between diploids and tetraploids and consequently may localize or function differently (Hollister et al., 2012; Yant et al., 2013). During prophase I in *A. arenosa,* immunolocalization of ASY1 is similar to that previously described in *A. thaliana* and *Brassica* (**Figure 1** ). ASY1 is initially observed as discrete punctate foci during G2 (**Figures 1A,G**), which extend and coalesce at leptotene to form a linear signal (**Figures 1B,H**) During zygotene and pachytene, the ASY1 signal is reduced in the ZYP1 labeled synapsed regions, reflecting either protein depletion or reorganization of the chromosome axes (**Figures 1C–F,I–L**). In *A. thaliana*, two duplicated tandem inverted genes, *ZYP1a* and *ZYP1b,* encode the synaptonemal complex transverse filament proteins (from here on referred to as ZYP1). ZYP1 is required for normal chromosome synapsis and promoting wild-type levels of crossovers. It is also necessary for resolving chromosome interlocks and preventing non-homologous crossovers (Higgins et al., 2005). In *A. arenosa*, ZYP1 also exhibits signatures of selection and strong differentiation between diploids and tetraploids and function or regulation could be affected (Hollister et al., 2012; Yant et al., 2013). In *A. thaliana*, the ZYP1 protein initially localizes to chromosomes as foci or short stretches during zygotene which then elongate until pachytene, when the chromosomes are fully synapsed (Higgins et al., 2005). In *A. arenosa *short stretches of ZYP1 are detected at early zygotene in both diploid and tetraploid (**Figures 1C,I**). By mid-zygotene the ZYP1 stretches become more extensive, accompanied by further synapsis initiation sites (**Figures 1D,J**). At this stage the tetraploid differs to the diploid as parallel linear ZYP1 stretches become apparent (**Figure 1J**, white arrows), which may reflect synchronous synapsis progression of both pairs of homologous chromosomes. During late-zygotene chromosome interlocks are visible in the tetraploid (**Figure 1K**), which are resolved by pachytene when discrete, condensed chromosomes are observed (**Figures 1F,L**). At pachytene, the synaptonemal complexes appear shorter in the tetraploid than the diploid which could be due to a difference in condensation as a result of amino acid changes described in Yant et al. (2013) or that the chromosomes are at different stages of condensation during pachytene (**Figures 1F,L**).

**FIGURE 2:**
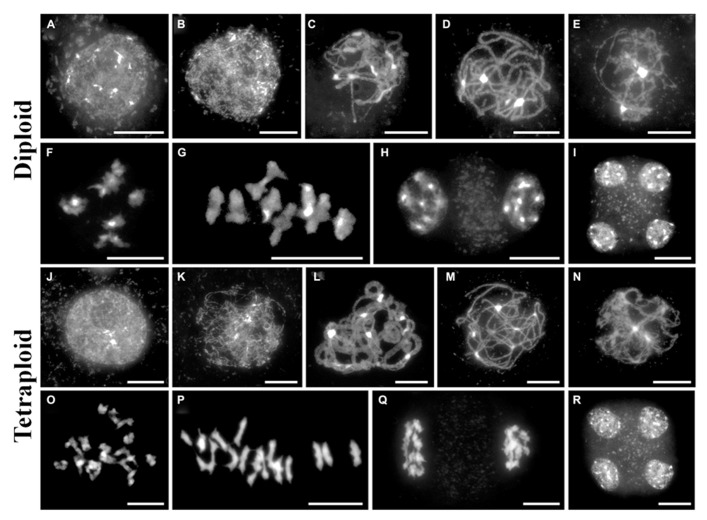
**A cytological meiotic atlas of diploid and tetraploid *Arabidopsis arenosa*.** **(A–I)** Representative meiotic stages of a diploid population collected from Strecno and a tetraploid population collected from Triberg **(J–R)**. G2 **(A,J)**, leptotene **(B,K)**, zygotene **(C,L)**, pachytene **(D,M)**, diplotene **(E,N)**, diakinesis **(F,O)**, metaphase I **(G,P)**, dyad **(H,Q)**, and tetrad **(I,R)**. Chromosomes have been stained with DAPI and bar = 10 μm.

A meiotic atlas of diploid and tetraploid *A. arenosa* is presented to be used as a cytological reference (**Figure 2**). Interestingly, it reveals an extra level of chromosome organization during zygotene in the tetraploid, compared to the diploid (**Figures 2C,L**). In the tetraploid, up to four chromosomes align and even greater numbers converge at the brightly DAPI stained heterochromatic regions. This organization has been observed in populations collected from Trencin, Slovakia and Triberg, Germany and may represent independent convergent adaptation or gene flow. It will be interesting to determine if these aligned chromosomes are pairs of homologs and if this is a mechanism which has evolved to prevent ectopic recombination. This may be consistent with the parallel linear ZYP1 stretches observed in **Figure 1J**.

**FIGURE 3:**
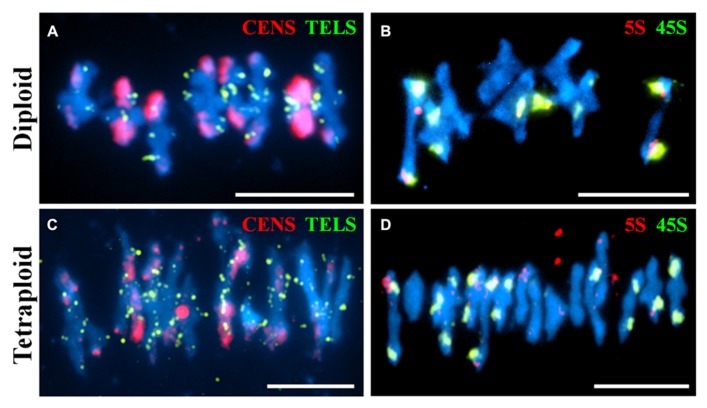
**Fluorescence *in situ *hybridization of meiotic chromosome spreads using repetitive DNA probes in diploid and tetraploid *Arabidopsis arenosa*.** Representative meiotic metaphase I stages in diploid **(A,B)** and tetraploid **(C,D)** using the centromere and telomere probes **(A,C)** and the 5S and 45S rDNA probes **(B,D)**. Chromosomes have been stained with DAPI (blue). Bar = 10 μm.

Fluorescence *in situ* hybridization is a useful cytological technique to label specific chromosomal regions. It may be used for identifying individual chromosomes, polymorphic loci or investigating the function of chromosomal regions in biological processes. We have used DNA probes to label repetitive DNA sequences (centromeres, telomeres, 5S and 45S ribosomal DNA) at metaphase I on diploid and tetraploid *A. arenosa* (**Figure 3**). At metaphase I it is possible to score chiasmata, the cytological sites of crossovers based on chromosome shape (Sanchez-Moran et al., 2001). The probes may be used to determine chiasma position, number and homologous or non-homologous associations in *A. arenosa*. The telomere probe has previously been used in *A. thaliana* (Armstrong et al., 2001) and the centromere probe in *A. arenosa* (Comai et al., 2003). Interestingly, the centromere repeat was highly polymorphic between and within chromosomes in the diploid (**Figure 3A**), which may reflect variations in centromere size or repetitive DNA element content. The telomeres are detected at the ends of the short and long chromosome arms (**Figures 3A,C**). In the diploid, the 5S and 45S rDNA distinguish the chromosomes into three groups: 1) three chromosomes with no probes; 2) two chromosomes with 45S only; 3) three chromosomes with 5S and 45S (**Figure 3B**). In the tetraploid, the probes are not always symmetrical revealing either previous chromosome rearrangements, insertions, deletions or non-homologous crossovers (**Figure 3D**). Non-homologous chiasmata have previously been reported in *A. thaliana* *ZYP1*^RNAi^ lines and the authors postulated that this may be due to the high sequence similarity in duplicated regions on non-homologous chromosomes (Higgins et al., 2005). It is conceivable that whole genome duplication in *A. arenosa* affects all aspects of meiotic surveillance, so that the obligate chiasma, homologous recombination, and interference may be disrupted.

## SUMMARY

In this chapter we have described cytological methods to investigate meiotic adaptation to polyploidy in *A. arenosa*. We have developed immunocytological techniques and used repetitive DNA probes for FISH. This has revealed differences between the diploid and tetraploid during meiosis. An extra level of chromosome organization appears to occur during zygotene in the tetraploid that does not occur in the diploid. In the tetraploid, multiple chromosomes align and associate at heterochromatic regions during zygotene as well as parallel ZYP1 stained chromosomes being observed. This is consistent with the bud sizes being greater in the tetraploid at this stage, indicating a possible delay to enable successful chromosome sorting and homologous recombination. However, in the tetraploid there are examples of bivalents at metaphase I with non-symmetrical 45S and 5S rDNA labeling may represent non-homologous crossovers, suggesting that *A. arenosa* has not yet evolved a completely stable solution to genome duplication.

## Conflict of Interest Statement

The authors declare that the research was conducted in the absence of any commercial or financial relationships that could be construed as a potential conflict of interest.

## AUTHOR CONTRIBUTIONS

James D. Higgins performed cytological experiments assisted by Kevin M. Wright and F. Chris H. Franklin. Kevin M. Wright and Kirsten Bomblies provided plant material and growth conditions. James D. Higgins, Kevin M. Wright, Kirsten Bomblies and F. Chris H. Franklin wrote the paper.

## Footnotes

1

2

3

4

5
